# Digital Connectedness in the Jackson Heart Study: Cross-sectional Study

**DOI:** 10.2196/37501

**Published:** 2022-11-21

**Authors:** Pramod Anugu, Md Abu Yusuf Ansari, Yuan-I Min, Emelia J Benjamin, Joanne Murabito, Karen Winters, Erica Turner, Adolfo Correa

**Affiliations:** 1 University of Mississippi Medical Center Jackson, MS United States; 2 Department of Data Science, School of Population Health University of Mississippi Medical Center Jackson, MS United States; 3 Boston University Boston, MA United States

**Keywords:** teleresearch, mobile technology, cardiovascular disease, Jackson Heart Study, mobile phone

## Abstract

**Background:**

Although new approaches for data collection, such as mobile technology and teleresearch, have demonstrated new opportunities for the conduct of more timely and less costly surveys in community-based studies, literature on the feasibility of conducing cardiovascular disease research using mobile health (mHealth) platforms among middle-aged and older African Americans has been limited.

**Objective:**

The purpose of this study was to contribute to the knowledge regarding the penetrance of internet and mobile technologies, such as cellphones or smartphones in existing large cohort studies of cardiovascular disease.

**Methods:**

A digital connectedness survey was conducted in the Jackson Heart Study (JHS), a Mississippi-based African American cohort study, as part of the annual follow-up calls with participants from July 2017 to February 2019.

**Results:**

Of the 4024 participants contacted, 2564 (63.7%) completed the survey. Among survey respondents, 2262 (88.2%) reported use of internet or cellphone, and 1593 (62.1%) had a smartphone. Compared to nonusers (n=302), internet or cellphone users (n=2262) were younger (mean age 80.1, SD 8.0 vs 68.2, SD 11.3 years), more likely to be affluent (n=778, 40.1% vs n=39, 15.4%), and had greater than high school education (n=1636, 72.5% vs n=85, 28.1%). Internet or cellphone users were less likely to have cardiovascular disease history compared to nonusers (136/2262, 6.6% vs 41/302, 15.8%). The prevalence of current smoking and average BMI were similar between internet or cellphone users and nonusers. Among internet or cellphone users, 1316 (58.3%) reported use of email, 504 (22.3%) reported use of apps to track or manage health, and 1269 (56.1%) expressed interest in using JHS-developed apps.

**Conclusions:**

Our findings suggest that it is feasible to use mHealth technologies to collect survey data among African Americans already enrolled in a longitudinal study. Our findings also highlight the need for more efforts to reduce the age and education divide in access and use of internet and smartphones for tracking health and research in African American communities.

## Introduction

Over decades, data collection in epidemiological longitudinal research into cardiovascular disease (CVD) has relied in part on costly and time-consuming surveys administered by phone or in person [1-4]. However, with the recent rapid growth in the use of digital or mobile technologies in the general population, there is now an opportunity to adopt eHealth or mobile health (mHealth) apps that are less expensive and more practical in research settings. mHealth as a part of eHealth can be defined as medical and public health practice supported by mobile devices, such as mobile phones, patient monitoring devices, personal digital assistants, and other wireless devices [5]. Over the past decades, mHealth app design and its role on the health behavior change among study participants has been discussed in several studies [6-8]. A qualitative study conducted on adults with type 1 diabetes showed that a well-designed mHealth app could serve to inform lifestyle choices, diabetes self-management tasks, preemptive self-care actions, and improved discussions with clinicians [9]. These evidences suggest that well-designed mHealth app can assist individuals especially older adults in independent living and self-management of (chronic) illnesses. mHealth apps have the prospect of participant convenience, collection of ‘real-world’ data, flexibility to pivot research agendas to focus on timely health issues (such as disparities in CVD and the long-term effects of COVID-19 pandemic), as well as the potential to be conducted in large and diverse populations. Although there have been recent efforts to examine mHealth apps for data collection in community-based studies [10,11], the literature on the feasibility of using mHealth applications in populations of middle-aged and older African Americans is limited.

The purpose of our study was to contribute to the knowledge regarding the penetrance of internet and mobile technologies, such as cellphones or smartphones in existing large cohort studies of CVD. Our study was modeled after a survey conducted in the Framingham Heart Study (FHS). The FHS cohort is a cohort of predominately White participants, largely based in New England; as such the findings from FHS may not be generalizable to other racial and ethnic groups or geographic regions. In this study, we conducted a digital connectedness survey in the Jackson Heart Study (JHS), a large cohort of exclusively middle-aged and older African Americans. We also examined the sociodemographic characteristics and CVD risk factors associated with digital connectedness. We have hypothesized that digitally connected individuals would be relatively younger, have higher educational attainment, and have an inverse association with CVD risk factor profiles compared to their counterparts who are not digitally connected.

## Methods

The JHS is a large prospective community-based observational study designed to investigate risk factors for CVD in African Americans. Details of the JHS study design, recruitment, and data collection have been described previously [12,13]. Briefly, 5306 African American participants residing in the Jackson metropolitan area in Mississippi were recruited for the study between 2000 and 2004. Three research center visits were conducted to date (visit 1 [baseline]: 2000-2004; visit 2: 2005-2008; and visit 3: 2009-2012). Additionally, the participants were contacted once a year by telephone (annual follow-up) to ascertain events and vital status as well as update health data and contact information.

### Ethical Considerations

All study participants provided written informed consent. The JHS was approved by the institutional review boards (Protocol 1998-6004) of Jackson State University, Tougaloo College, the Mississippi State Department of Health, and the University of Mississippi Medical Center.

### Digital Connectedness Survey

All living JHS participants contacted for the annual follow-up calls between July 2017 and February 2019 were invited to participate in a digital connectedness survey. The survey collected data on the use of internet, cellphone, smartphone, email, health apps, social media, and computer games. Additionally, smartphone users were asked whether they had any software apps that helped them track or manage health and about the types of health apps they had. They were also asked about any technology that was used to store health readings digitally.

### Sociodemographic Characteristics and CVD Risk Factors

Sociodemographic characteristics examined in this study included sex, age, smoking status, BMI, education, and income, collected at the baseline (visit 1). CVD risk factors examined included diabetes, hypertension, hyperlipidemia, CVD history, collected at visit 3. Participants’ age was calculated at the time of the survey. Age was grouped into the following 4 levels: <65; ≥65 to < 75; ≥75 to <85; and ≥85 years. Education was categorized as follows: “less than high school”; “high school graduate or Graduate Education Development certificate”; and “attended vocational school, trade school, or college.” Income was categorized into 4 groups according to the US census poverty levels based on household income and family size. The categories were assigned as “poor,” representing income lower than poverty level; “lower-middle,” representing income 1 to 1.5 times the poverty level; “upper-middle,” representing income >1.5 but <3.5 times the poverty level; and “affluent,” representing income ≥3.5 times the poverty level [14,15]. Diabetes was defined based on the following: fasting glucose ≥126 mg/dL; HbA1c ≥6.5%; use of diabetes medication; or self-reported diabetes [16]. Hypertension was defined as blood pressure ≥140/90 mm Hg (per the seventh report of the Joint National Committee) or use of blood pressure lowering medication [17]. Hyperlipidemia was defined as total fasting cholesterol ≥200 mg/dL or taking any statin medication.

### Classification of Digital Connectedness and Participant Characteristics

For the purpose of this study, we defined digital connectedness as use of internet and cellphone; specifically, respondents who gave affirmative responses to the questions “Do you use the internet at least occasionally?” or “Do you use a cell phone?” were considered digitally connected. A negative response to both questions classified the respondents as not digitally connected. Instead of smartphone users, combining cellphone users and the internet users would allow us to connect with a larger cohort of African American population.

### Statistical Analysis

All categorical data were reported as counts and relative frequencies as percentages. Continuous data were presented as mean (SD). We compared the characteristics between respondents and nonrespondents of the digital connectedness survey using logistic regression models adjusted for age and sex. We compared the sociodemographic characteristics and CVD risk factors between users and nonusers of internet or cellphone, using logistic regression models adjusted for age and sex. The associations between CVD risk factors and internet and cellphone use were reported as adjusted odds ratios (AORs) and 95% CIs. A 2-sided *P* value <.05 was considered statistically significant for all analyses. All statistical analyses were performed using R statistical software (version 4.0.2; The R Foundation for Statistical Computing).

## Results

Of the 4024 JHS participants contacted, 2564 (63.7%) completed the survey. The mean age of the respondents was 69.6 (SD 11.6) years, and 64.1% (1644/2564) of them were female. Compared to the nonrespondents, respondents were older (mean age 69.6, SD11.6 years vs mean age 64.8, SD 12.3 years; *P*<.001); more likely to have completed less than high school or attended vocational school, trade school, or college (1721/2564, 67.3% vs 972/1460, 66.8%; overall *P*<.001); more affluent (817/2194, 37.2% vs 324/1216, 26.6%; overall *P*<.001); and less likely to be a current smoker (267/2545, 10.5% vs 207/1445, 14.3%; *P*=.002; Table S1 in Multimedia Appendix 1). Among the 2564 respondents, 2262 (88.2%) were users of internet or cellphone (internet users 1507, 58.9%; cellphone users 2230, 87.1%). Use of a smartphone was reported in 1593 (62.1%) respondents.

### Comparison of Sociodemographic Characteristics and CVD Risk Factors Between Internet or Cellphone Users and Nonusers

**Figure 1:**
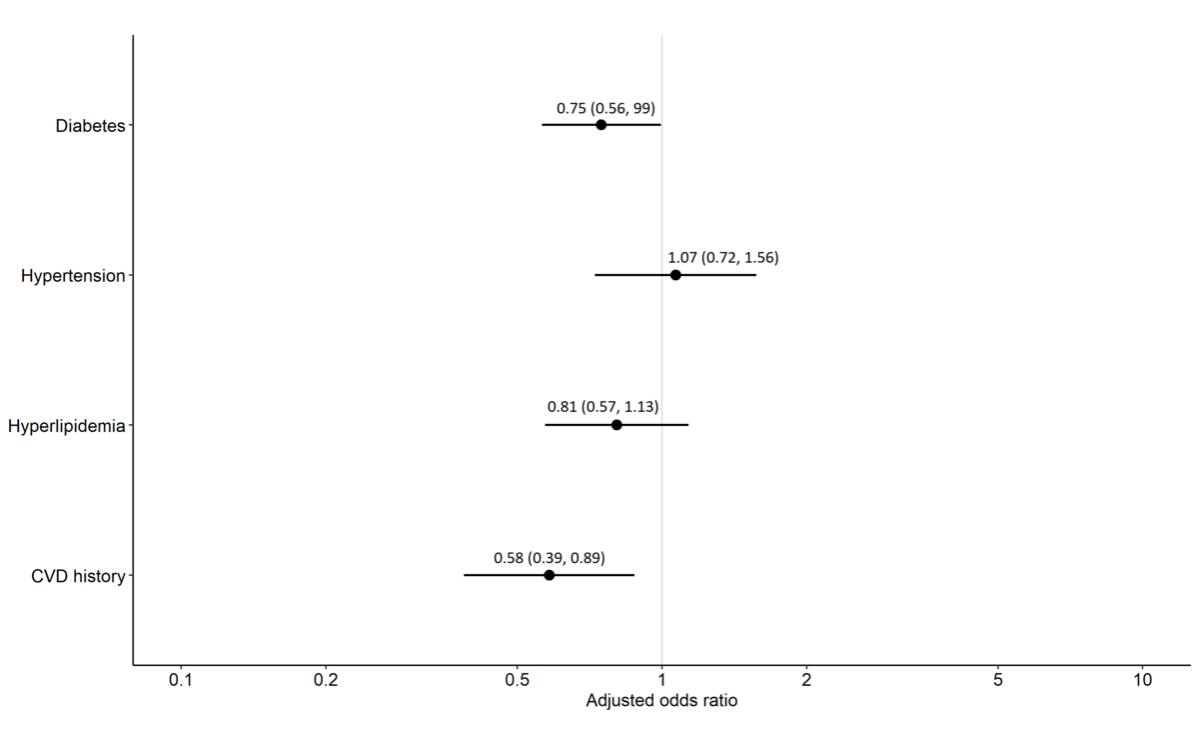
Adjusted odds ratio (95% CI) of cardiovascular disease (CVD) risk factors by internet or cellphone users versus nonusers. Odd ratios are age- and sex-adjusted using logistic regression models. Vertical line represents null effect.

Compared to nonusers, internet and cellphone users were younger (mean age 68.2, SD 11.3 years vs mean age 80.1, SD 8.0 years; *P*<.001); they were more likely to have attended vocational school, trade school, or college (1636/2262, 72.5% vs 85/302, 28.1%; overall *P*<.001); have higher income (affluent 778/2262, 40.1% vs 39/302, 15.4%; overall *P*<.001); and be employed (716/2262, 31.7% vs 7/302, 2.3%; overall *P*<.001; Table 1). Internet or cellphone users were less likely to have a history of CVD (136/2262, 6.6% vs 41/302, 15.8%; AOR 0.58, 95% CI 0.39-0.89) and diabetes (624//2262, 30.3% vs 108/302, 41.7%; AOR 0.75; 95% CI 0.56-0.99) compared to nonusers (Table 1; Figure 1). There was no difference in sex distributions between users and nonusers of internet or cellphone. The prevalence of current smoking, hypertension, hyperlipidemia, and average BMI were similar in internet or cellphone users and nonusers. Similar patterns were observed comparing users and nonusers of internet, cellphone, and smartphone (Table S2 in Multimedia Appendix 1).

**Table 1:** Sociodemographic characteristics and cardiovascular disease (CVD) risk factors by internet or cellphone use among digital connectedness survey respondents.

Characteristics				Internet or cellphone use, n (%)					
				Nonuser, n=302 (11.8)		User, n=2262 (88.2)		*P* value^a^	
**Sex, n (%)**									.11
	Female		220 (72.8)		1424 (63)				
	Male		82 (27.2)		838 (37)				
Age (years)^b^, mean (SD)				80.1 (8.0)		68.2 (11.3)		<.001	
**Age (years), n (%)**									<.001
		<65	13 (4.3)		864 (38.2)				
		≥65 to <75	51 (16.9)		666 (29.4)				
		≥75 to <85	150 (49.7)		601 (26.6)				
		≥85	88 (29.1)		131 (5.8)				
**Education, n (%)**									<.001
	Less than high school		88 (29.1)		389 (17.2)				
	High school graduate or GED^c^		129 (42.7)		232 (10.3)				
	Attended vocational school or trade school		85 (28.1)		1636 (72.5)				
**Income, n (%)**									<.001
		Poor	55 (21.7)		175 (9)				
		Lower-middle	95 (37.5)		357 (18.4)				
		Upper-middle	64 (25.3)		631 (32.5)				
		Affluent	39 (15.4)		778 (40.1)				
Diabetes, n (%)				108 (41.7)		624 (30.3)		.05	
Hypertension, n (%)				219 (84.6)		1504 (72.6)		.73	
Hyperlipidemia, n (%)				207 (79.9)		1416 (68.3)		.22	
Current smoker, n (%)				31 (10.3)		236 (10.5)		.99	
BMI (kg/m^2^), mean (SD)				31.2 (6.8)		32.1 (6.7)		.48	
Cardiovascular disease history, n (%)				41 (15.8)		136 (6.6)		.01	
**Employed, n (%)**									<.001
		Employed	7 (2.3)		716 (31.7)				
		Homemaking	1 (0.3)		7 (0.3)				
		Retired	285 (94.4)		1423 (63)				
		Unemployed	9 (2.9)		114 (5)				

^a^*P* values are age- and sex-adjusted using logistic regression models.

^b^Age at the time of digital connectedness survey.

^c^GED: Graduate Education Development.

### Experience with the Use of Technologies (Apps) Among Internet or Cellphone Users

Among the 2262 internet or cellphone users, 1316 (58.3%) reported use of email; 504 (22.3%) reported use of software applications to track or manage health; and 1565 (69.2%) reported use of digital health technology, which stores health readings digitally (Table 2). Using social media to keep in touch with friends and family was reported among 830 (36.7%) internet or cellphone users, and 797 (35.5%) internet or cellphone users reported playing games on their devices.

When asked about whether they would be interested in using a JHS-developed mobile phone app to respond to health questions, 1269/2262 (56.1%) internet or cellphone users expressed interest, 353 (15.6%) expressed possible interest (by answering “not sure” or “maybe”), and 640 (28.3%) expressed no interest. Internet or cellphone users were significantly more inclined toward using JHS-developed apps as compared to the nonusers (1269/2262, 56.1% vs 14/302, 4.6%; *P*<.001).

**Table 2:** Experiences with technology or apps use among internet or cellphone users (N=2262).

Internet or cellphone users		Values, n (%)
**Email, n=1316 (58.3)**		
	Can read new email	1316 (58.3)
	Can use the reply feature	981 (43.4)
	Can send an email	1001 (44.3)
	Can open a file attached to an email	842 (37.2)
**Apps to track health or manage health, n=504 (22.3)**		
	Exercise, fitness, pedometer, or heart rate monitor	484 (21.4)
	Diet, food, calorie counter	46 (2)
	Blood pressure	16 (0.7)
	Weight	23 (1)
	Blood sugar or diabetes	7 (0.3)
	Medication management	7 (0.3)
	Sleep	4 (0.2)
	Mood	7 (0.3)
	Other	6 (0.3)
**Digital health technology, n=1565 (69.2)**		
	Digital blood pressure cuff	1387 (61.3)
	Digital scale	549 (24.3)
	Digital glucometer	418 (18.5)
	Other	3 (0.1)
**Interest in using the JHS^a^ app in the future, n=2262 (100)**		
	Yes	1269 (56.1)
	No	640 (28.3)
	Not sure or maybe	353 (15.6)
**Social media, n=830 (36.7)**		
	Facebook	784 (34.7)
	LinkedIn	94 (4.2)
	Google Plus	46 (2.0)
	Twitter	83 (3.7)
	Other	61 (2.7)
**Playing games, n=797 (35.5)**		
	On computer	270 (11.9)
	On tablet	337 (14.9)
	On smartphone	517 (22.9)
	On video game console	37 (1.6)
	On other devices	5 (0.2)

^a^JHS: Jackson Health Study.

## Discussion

### Principal Findings

Overall, 63.7% of the JHS participants surveyed responded to our digital connectedness survey. Compared to nonrespondents, survey respondents were older, more affluent, less likely to be current smokers, and less likely to have a history of CVD. Among survey respondents, close to 90% were users of internet and cellphone, and over half of them had a smartphone. Among users of internet or cellphone, close to 60% reported use of email, over 20% had used software apps to track or manage health and reported use of social media or played games on their devices, and over 70% expressed interest or possible interest in using an app to answer health questions. Participants interest for using a JHS-developed app was encouraging. We can integrate the app as an additional alternative tool for data collection. This could lead us to lower the participants’ burden. We can receive ratings and reviews within the app from the app users to determine the success and possible places for improvement.

### Strengths and Limitations

An important strength of our survey is that it is based on a community-based cohort of middle-aged and older African American men and women residents of Jackson, Mississippi who have been active participants in annual follow-up interviews in a longitudinal research study (ie, the JHS). Another strength is the high level of participation achieved, which probably reflects a high level of trust and commitment of participants with the JHS. Our survey also has some limitations. First, the survey was conducted on middle-aged to older adult African American residents of Jackson, Mississippi, and participants in the JHS. As such, the generalizability of our survey findings to other age groups, geographic regions, and race or ethnicities is uncertain. Second, the study was completed in 2019; it may not reflect the current prevalence of internet or cellphone use, which may have increased over time and thus may limit generalizations to more contemporary settings. Also, the sociodemographic characteristics used from visit 1 may not necessarily represent the characteristics of the participants in 2019. Third, although most JHS participants reported access to internet or cellphone, the extent to which they would use internet or cellphone to respond to research surveys or for ongoing health surveillance remains to be determined. Moreover, using cellphones for research requires participants’ contact information to be updated regularly. In addition to that, the extent to which African Americans enrolled in a new community-based research project would participate in mHealth remains to be determined.

Lastly, there are potential risks to African American community arising from the promotion of secondary (and often commercial) uses of participant data; discriminatory profiling and inaccurate health status notifications because of algorithmic issues may undermine the trust in this new form of research [18,19].

Digital collection, monitoring, and transmission of data always involve some challenges related to data security and privacy that are often different from paper-based surveys. To avoid any security risk, a rigorous encryption policy needs to be employed. In JHS, the storage devices are bitLocker encrypted, with advanced encryption standard 256 enabled. Secure Sockets Layer (Transport Layer Security 1.2) certificate is installed and enforced. A natural barrier to using mHealth technologies is linked to physical limitation, which relates to the older age groups and comorbidities among the participants. Any attempt to design mHealth apps for older age groups needs to address motivational and cognitive barriers to older adults [20]. More research needs to be done to identify the reasons why certain populations such as older age groups in African American communities do not use mHealth technologies and if there are any improvements on using mHealth technologies that lead to ease of data collection and better CVD outcomes.

### Comparisons With Prior Work

Although a high proportion of our survey participants reported use of internet or cellphone, the proportion observed is somewhat lower than national estimates. According to a 2021 survey conducted by the Pew Research Center [21], the vast majority of Americans (97%) own a cellphone and 85% own a smartphone, with no substantial differences among Black and White populations. However, among Americans who are 65 years and older, these percentages were lower (92% reported owning a cellphone and 61% owned a smartphone). Thus, one possible explanation for a lower proportion of our survey participants reporting use of internet and cell phone compared to national estimates may be due to the older age of our survey participants. Two other surveys conducted in 2019 by Pew Research Center [22,23] found that the digital divide persisted between rural and nonrural America, impacted by income levels [22,23]; therefore, the lower proportion of our survey participants reporting use of internet or cellphone may also reflect some differences in employment and income levels. Use of a smartphone may bridge this digital divide, as it offers a less expensive yet diverse alternative to the in-home Wi-Fi connection. It also provides more internet access to the undeserved communities compared to in-home Wi-Fi connection [24,25].

The digital health trend has the potential to transform health care [26-30]. The eHealth and mHealth technologies have come along as convenient and efficient ways to collect daily or frequent health data from individual persons to inform clinical care and research. For instance, several eHealth and mHealth studies using digital technologies had been piloted and tested in the FHS [31-36]. Nevertheless, the collection of mHealth information is relatively new and remains less tested in established population-based African American cohorts and clinical research. Some of the challenges for collecting health information remotely that need to be addressed include high attrition rates and lack of representativeness of the target populations [37]. Additionally, African Americans have not been well represented in these clinical and research studies, raising the question as to the feasibility of mHealth research in African American communities. Our survey findings among participants in the JHS suggest that it is feasible to conduct CVD research using mHealth platforms in an African American community in the southern part of the United States.

During the COVID-19 pandemic, in-person interviews in cohort studies was difficult to conduct, and that highlighted the need for more practical remote data collection methods. Although our survey was completed well before the pandemic had started (ie, February 2019), we were able to connect with a large group of respondents among which almost 90% were digitally connected. This experience suggests that JHS cohort participants can be reached digitally in the future when a face-to-face interview may not be possible. Use of cellphone and smartphone technology may increase the efficiency of research studies, may provide more frequent and ‘real-world’ data on participants, and may allow participants greater choice and convenience regarding when and where to respond to research surveillance.

### Conclusions

Our survey findings suggest that it is feasible to collect survey data using eHealth and mHealth technologies among middle-aged and older African Americans already enrolled in an ongoing research study in Jackson, Mississippi. Our findings also highlight the need for more efforts to reduce the age and education divide in access and use of internet and smartphones for tracking health and research in African American communities.

## Appendix

Multimedia Appendix 1 Supplementary tables.

Multimedia Appendix 2 Questionnaire Form.

## Abbreviations

AOR: adjusted odds ratio
CVD: cardiovascular disease
FHS: Framingham Heart Study
JHS: Jackson Heart Study
mHealth: mobile health

## References

[1] Khoury M, Lam T, Ioannidis J (2013). Transforming epidemiology for 21st century medicine and public health. Cancer Epidemiol Biomarkers Prev.

[2] Lauer MS (2012). Time for a creative transformation of epidemiology in the United States. JAMA.

[3] Rosbash M (2011). A threat to medical innovation. Science.

[4] Sorlie PD, Bild DE, Lauer MS (2012). Cardiovascular epidemiology in a changing world--challenges to investigators and the National Heart, Lung, and Blood Institute. Am J Epidemiol.

[5] WHO mHealth: new horizons for health through mobile technologies: second global survey on eHealth. World Health Organization.

[6] Bhuyan SS, Lu N, Chandak A, Kim H, Wyant D, Bhatt J, Kedia S, Chang CF (2016). Use of mobile health applications for health-seeking behavior among US adults. J Med Syst.

[7] Vollmer DD, Fair K, Hong YA, Beaudoin CE, Pulczinski J, Ory MG (2015). Apps seeking theories: results of a study on the use of health behavior change theories in cancer survivorship mobile apps. JMIR Mhealth Uhealth.

[8] McCurdie T, Taneva S, Casselman M, Yeung M, McDaniel C, Ho W, Cafazzo J (2012). mHealth consumer apps: the case for user-centered design. Biomed Instrum Technol.

[9] Ritholz MD, Henn O, Atakov Castillo A, Wolpert H, Edwards S, Fisher L, Toschi E (2019). Experiences of adults with type 1 diabetes using glucose sensor–based mobile technology for glycemic variability: qualitative study. JMIR Diabetes.

[10] Fox CS, Hwang S, Nieto K, Valentino M, Mutalik K, Massaro JM, Benjamin EJ, Murabito JM (2016). Digital Connectedness in the Framingham Heart Study. JAHA.

[11] Schrauben SJ, Appel L, Rivera E, Lora CM, Lash JP, Chen J, Hamm LL, Fink JC, Go AS, Townsend RR, Deo R, Dember LM, Feldman HI, Diamantidis CJ, CRIC Study Investigators (2021). Mobile health (mHealth) technology: assessment of availability, acceptability, and use in CKD. Am J Kidney Dis.

[12] Sempos CT, Bild DE, Manolio TA (1999). Overview of the Jackson Heart Study: a study of cardiovascular diseases in African American men and women. Am J Med Sci.

[13] Taylor H (2005). The Jackson Heart Study: an overview. Ethn Dis.

[14] Carpenter MA, Crow R, Steffes M, Rock W, Heilbraun Jeffrey, Evans Gregory, Skelton Thomas, Jensen R, Sarpong D (2004). Laboratory, reading center, and coordinating center data management methods in the Jackson Heart Study. Am J Med Sci.

[15] Min Y, Anugu P, Butler KR, Hartley TA, Mwasongwe S, Norwood AF, Sims M, Wang W, Winters KP, Correa A (2017). Cardiovascular disease burden and socioeconomic correlates: findings from the Jackson Heart Study. JAHA.

[16] American DA (2010). Diagnosis and classification of diabetes mellitus. Diabetes Care.

[17] Chobanian AV, Bakris GL, Black HR, Cushman WC, Green LA, Izzo JL, Jones DW, Materson BJ, Oparil S, Wright JT, Roccella EJ, Joint NCOPDEOHBPNHLI, National HBPEPCC (2003). Seventh report of the joint national committee on prevention, detection, evaluation, and treatment of high blood pressure. Hypertension.

[18] Callier S, Fullerton SM (2020). Diversity and inclusion in unregulated mhealth research: addressing the risks. J Law Med Ethics.

[19] Nebeker C, Murray K, Holub C, Haughton J, Arredondo EM (2017). Acceptance of mobile health in communities underrepresented in biomedical research: barriers and ethical considerations for scientists. JMIR Mhealth Uhealth.

[20] Wildenbos GA, Jaspers MWM, Schijven MP, Dusseljee-Peute LW (2019). Mobile health for older adult patients: using an aging barriers framework to classify usability problems. Int J Med Inform.

[21] (2021). Mobile fact sheet. Pew Research Center.

[22] Center P (2019). Digital gap between rural and nonrural America persists. Some digital divides persist between rural, urban and suburban America.

[23] Vogels EA (2021). Digital divide persists even as lower-income Americans make gains in tech adoption. Pew Research Center.

[24] (2015). Chapter one: a portrait of smartphone ownership. Pew Research Center.

[25] Smith A, Zickhur K (2012). Digital differences. Pew Research Center.

[26] Patel MS, Asch DA, Volpp KG (2015). Wearable devices as facilitators, not drivers, of health behavior change. JAMA.

[27] Powell AC, Landman AB, Bates DW (2014). In search of a few good apps. JAMA.

[28] Steinhubl SR, Muse ED, Topol EJ (2013). Can mobile health technologies transform health care?. JAMA.

[29] Topol EJ, Steinhubl SR, Torkamani A (2015). Digital medical tools and sensors. JAMA.

[30] Smith A (2015). U.S. Smartphone use in 2015. Pew Research Center.

[31] McManus DD, Trinquart L, Benjamin EJ, Manders ES, Fusco K, Jung LS, Spartano NL, Kheterpal V, Nowak C, Sardana M, Murabito JM (2019). Design and preliminary findings from a new electronic cohort embedded in the framingham heart study. J Med Internet Res.

[32] Lin H, Sardana M, Zhang Y, Liu C, Trinquart L, Benjamin EJ, Manders ES, Fusco K, Kornej J, Hammond MM, Spartano NL, Pathiravasan CH, Kheterpal V, Nowak C, Borrelli B, Murabito JM, McManus DD (2020). Association of habitual physical activity with cardiovascular disease risk. Circ Res.

[33] Pathiravasan CH, Zhang Y, Trinquart L, Benjamin EJ, Borrelli B, McManus DD, Kheterpal V, Lin H, Sardana M, Hammond MM, Spartano NL, Dunn AL, Schramm E, Nowak C, Manders ES, Liu H, Kornej J, Liu C, Murabito JM (2021). Adherence of mobile app-based surveys and comparison with traditional surveys: ecohort study. J Med Internet Res.

[34] Patel MS, Benjamin EJ, Volpp KG, Fox CS, Small DS, Massaro JM, Lee JJ, Hilbert V, Valentino M, Taylor DH, Manders ES, Mutalik K, Zhu J, Wang W, Murabito JM (2017). Effect of a game-based intervention designed to enhance social incentives to increase physical activity among families: the be fit randomized clinical trial. JAMA Intern Med.

[35] Spartano NL, Lin H, Sun F, Lunetta KL, Trinquart L, Valentino M, Manders ES, Pletcher MJ, Marcus GM, McManus DD, Benjamin EJ, Fox CS, Olgin JE, Murabito JM (2019). Comparison of on-site versus remote mobile device support in the framingham heart study using the health eheart study for digital follow-up: randomized pilot study set within an observational study design. JMIR Mhealth Uhealth.

[36] Trinquart L, McManus D, Nowak C (2019). Abstract 13084: increasing engagement in the electronic Framingham Heart Study (eFHS): a factorial randomized trial. Circulation.

[37] Pratap A, Neto EC, Snyder P, Stepnowsky C, Elhadad N, Grant D, Mohebbi MH, Mooney S, Suver C, Wilbanks J, Mangravite L, Heagerty PJ, Areán Pat, Omberg L (2020). Indicators of retention in remote digital health studies: a cross-study evaluation of 100,000 participants. NPJ Digit Med.

