# Supplementary material for: Digital Connectedness in the Jackson Heart Study: Cross-sectional Study
Source: J Med Internet Res. 2022 Nov 21;24(11):e37501. doi: 10.2196/37501 (PMC9723970; doi:10.2196/37501)
Supplement: Multimedia Appendix 1 [file jmir_v24i11e37501_app1.docx]

**Table S1.** Sociodemographic Characteristics and CVD Risk Factors among Jackson Heart Study (JHS) Participants by Response Status to the Digital Connectedness Survey.

|  | **Digital Connectedness Survey** | |  |
| --- | --- | --- | --- |
|  | **Non-respondent**  N=1460 (36.3%) | **Respondent**  N = 2564 (63.7%) | ***P*** **value†** |
| **Sex, n (%)** |  |  | .14 |
| Female | 946 (64.8) | 1644 (64.1) |  |
| Male | 514 (35.2) | 920 (35.9) |  |
| **Age, years*, mean (SD)** | 64.8 (12.3) | 69.6 (11.6) | <.001 |
| **Age, years*, n (%)** |  |  | <.001 |
| < 65 | 810 (55.5) | 877 (34.2) |  |
| ≥ 65 to < 75 | 328 (22.5) | 717 (28.0) |  |
| ≥ 75 to < 85 | 212 (14.5) | 751 (29.3) |  |
| ≥ 85 | 110 (7.5) | 219 (8.54) |  |
| **Education, n (%)** |  |  | <.001 |
| Less than high school | 185 (12.7) | 361 (14.1) |  |
| High school graduate/GED | 299 (20.5) | 477 (18.6) |  |
| Attended vocational school, trade school | 972 (66.8) | 1721 (67.3) |  |
| **Income, n (%)** |  |  | <.001 |
| Poor | 207 (17.0) | 230 (10.5) |  |
| Lower-middle | 295 (24.3) | 452 (20.6) |  |
| Upper-middle | 390 (32.1) | 695 (31.7) |  |
| Affluent | 324 (26.6) | 817 (37.2) |  |
| **Diabetes, n (%)** | 298 (29.2) | 732 (31.6) | .69 |
| **Hypertension, n (%)** | 702 (68.3) | 1723 (73.9) | .96 |
| **Hyperlipidemia, n (%)** | 626 (60.9) | 1623 (69.6) | .01 |
| **Current smoker, n (%)** | 207 (14.3) | 267 (10.5) | .002 |
| **BMI, kg/m^2^, mean (SD)** | 32.9 (7.9) | 32.0 (6.8) | .09 |
| **CVD history, n (%)** | 83 (8.1) | 177 (7.6) | .03 |
| **Employed, n (%)** |  |  | .02 |
| Employed | 17 (51.5) | 723 (28.2) |  |
| Homemaking | 0 (0.0) | 8 (0.3) |  |
| Retired | 14 (42.4) | 1708 (66.7) |  |
| Unemployed | 2 (6.1) | 123 (4.8) |  |
| *Age at the time of Digital Connectedness Survey for respondents; age at January 2019 for non-respondents.  BMI = Body mass index; CVD = Cardiovascular disease.  ^†^*P* values are age- and sex-adjusted using logistic regression models. | | | |

**Table S2.** Sociodemographic Characteristics and CVD Risk Factors among Digital Connectedness Survey Respondents by Internet, Cellphone and Smartphone Use.

|  | **No internet** | **Internet** | ***P*** **value^†^** | **No cellphone** | **Cellphone** | ***P*** **value^†^** | **No smartphone** | **Smartphone** | ***P* value^†^** |
| --- | --- | --- | --- | --- | --- | --- | --- | --- | --- |
|  | **N=1053 (41.1%)** | **N=1507 (58.9%)** |  | **N=331 (12.9%)** | **N=2230 (87.1%)** |  | **N=593 (23.1%)** | **N=1593 (62.1%)** |  |
| **Sex, n (%)** |  |  | .13 |  |  | .04 |  |  | .43 |
| Female | 693 (65.8) | 949 (63.0) |  | 243 (73.4) | 1400 (62.8) |  | 396 (66.8) | 977 (61.3) |  |
| Male | 360 (34.2) | 558 (37.0) |  | 88 (26.6) | 830 (37.2) |  | 197 (33.2) | 616 (38.7) |  |
| **Age, years*, mean (SD)** | 76.9 (8.8) | 64.5 (10.5) | <.001 | 79.4 (8.5) | 68.1 (11.3) | <.001 | 76.0 (8.5) | 65.3 (10.7) | <.001 |
| **Age, years*, n (%)** |  |  | <.001 |  |  | <.001 |  |  | <.001 |
| < 65 | 108 (10.3) | 769 (51.0) |  | 23 (6.9) | 854 (38.3) |  | 67 (11.3) | 764 (48.0) |  |
| ≥ 65 to < 75 | 245 (23.3) | 470 (31.2) |  | 60 (18.1) | 657 (29.5) |  | 153 (25.8) | 495 (31.1) |  |
| ≥ 75 to < 85 | 514 (48.8) | 235 (15.6) |  | 155 (46.8) | 593 (26.6) |  | 294 (49.6) | 289 (18.1) |  |
| ≥ 85 | 186 (17.7) | 33 (2.2) |  | 93 (28.1) | 126 (5.6) |  | 79 (13.3) | 45 (2.8) |  |
| **Education, n (%)** |  |  | <.001 |  |  | <.001 |  |  | <.001 |
| Less than high school | 313 (29.8) | 47 (3.12) |  | 132 (39.9) | 229 (10.3) |  | 146 (24.8) | 81 (5.1) |  |
| High school graduate/GED | 305 (29.1) | 171 (11.4) |  | 91 (27.5) | 386 (17.3) |  | 155 (26.4) | 222 (13.9) |  |
| Attended vocational school, trade school | 431 (41.1) | 1288 (85.5) |  | 108 (32.6) | 1610 (72.4) |  | 287 (48.8) | 1290 (81.0) |  |
| **Income, n (%)** |  |  | <.001 |  |  | <.001 |  |  | <.001 |
| Poor | 152 (17.4) | 78 (5.9) |  | 57 (20.3) | 173 (9.1) |  | 72 (14.5) | 98 (7.1) |  |
| Lower-middle | 269 (30.7) | 181 (13.8) |  | 103 (36.7) | 349 (18.3) |  | 141 (28.5) | 199 (14.5) |  |
| Upper-middle | 262 (29.9) | 431 (32.8) |  | 72 (25.6) | 622 (32.5) |  | 149 (30.1) | 458 (33.3) |  |
| Affluent | 193 (22.0) | 624 (47.5) |  | 49 (17.4) | 767 (40.1) |  | 133 (26.9) | 622 (45.2) |  |
| **Diabetes, n (%)** | 357 (38.3) | 374 (27.0) | .30 | 115 (40.6) | 616 (30.3) | .09 | 192 (36.0) | 415 (28.4) | .46 |
| **Hypertension, n (%)** | 777 (83.2) | 942 (67.6) | .37 | 238 (84.1) | 1483 (72.5) | .76 | 441 (82.6) | 1010 (68.6) | .69 |
| **Hyperlipidemia, n (%)** | 710 (76.0) | 911 (65.4) | .68 | 222 (78.4) | 1398 (68.3) | .45 | 393 (73.6) | 980 (66.5) | .63 |
| **Current smoker, n (%)** | 133 (12.7) | 133 (8.9) | .01 | 35 (10.7) | 232 (10.5) | .05 | 65 (11.1) | 164 (10.4) | .04 |
| **BMI, kg/m^2^, mean (SD)** | 31.5 (6.6) | 32.3 (6.8) | .11 | 31.4 (6.9) | 32.0 (6.7) | .34 | 31.8 (6.6) | 32.1 (6.7) | .01 |
| **CVD history, n (%)** | 97 (10.4) | 79 (5.7) | .97 | 42 (14.8) | 135 (6.6) | .02 | 48 (8.9) | 86 (5.8) | .56 |
| **Employed, n (%)** |  |  | <.001 |  |  | <.001 |  |  | <.001 |
| Employed | 70 (6.7) | 653 (43.4) |  | 11 (3.3) | 712 (32.0) |  | 49 (8.2) | 643 (40.4) |  |
| Homemaking | 5 (0.5) | 3 (0.2) |  | 1 (0.3) | 7 (0.3) |  | 3 (0.5) | 3 (0.2) |  |
| Retired | 933 (88.6) | 771 (51.2) |  | 308 (93.1) | 1397 (62.7) |  | 520 (87.7) | 856 (53.8) |  |
| Unemployed | 45 (4.3) | 78 (5.2) |  | 11 (3.3) | 112 (5.0) |  | 21 (3.5) | 89 (5.6) |  |
| *Age at the time of Digital Connectedness Survey.  BMI = Body mass index; CVD = Cardiovascular disease.  ^†^*P* values are age- and sex-adjusted using logistic regression models. | | | | | | | | | |
