# Supplementary material for: Digital Connectedness in the Jackson Heart Study: Cross-sectional Study
Source: J Med Internet Res. 2022 Nov 21;24(11):e37501. doi: 10.2196/37501 (PMC9723970; doi:10.2196/37501)
Supplement: Multimedia Appendix 2 [file jmir_v24i11e37501_app2.pdf]

Jackson Heart Study Electronic Device Survey

ID NUMBER ______________________________

Contact Year ______________________________

LAST NAME ______________________________

INITIALS ______________________________

A. INTERNET

Jackson Heart Study investigators are thinking about using new electronic tools to conduct research on your heart and general health. We are interested in finding out what kind of electronic tools you use, including computers, internet and cell phones.

This survey will take about 5 minutes. You will have to finish the survey in one sitting. You will not be able to save it and come back later.

You do not have to complete this survey if you do not wish to do so.

We want you to feel comfortable filling out this and future forms online. Please note that data provided via this survey are secure and confidential. These data will be used for research purposes only.

1. Do you use the internet at least occasionally?

O Yes

O No → **GO TO QUESTION 4**

1a. How do you access the internet? (check all that apply)

☐ Smart phone

☐ Tablet

☐ Computer

2. On average, how many hours per day do you use the internet?

____

(number of hours)

3. Do you have wireless internet ("wi-fi") in your home?

o Yes
o No → **GO TO QUESTION 4**

B. EMAIL

4. Do you use email?

o Yes
o No → **GO TO QUESTION 6**

5. Can you… (check all that apply)

☐ Read new emails?
☐ Use the reply feature?
☐ Send an email?
☐ Open a file attached to an email?

C. CELL PHONES

6. Do you use a cell phone?

o Yes
o No→ **GO TO QUESTION 12**

7. Some cell phones are called "smartphones" because of certain features they have. Is your cell phone a smartphone, such as an iPhone, Android?

o Yes
o No→ **GO TO QUESTION 12**

8. What type of smart phone do you use? (check all that apply)

☐ iPhone
☐ Android-based phone (e.g. Samsung, HTC, Google, Motorola, LG)
☐ Other (describe below)

8a. Please describe OTHER cell phone:

____

9. Please tell us if you ever use your cell phone to do any of the following things: (check all that apply)

☐ Send or receive email
☐ Send or receive text messages
☐ Take a picture
☐ Access the internet
☐ None of the above

10. On your phone, do you have any software applications or "apps" that help you track or manage your health?

o Yes
o No→ **GO TO QUESTION 12**

11. Health apps you currently have on your phone... (check all that apply)

☐ Exercise, fitness, pedometer or heart rate monitor (includes specific types of exercise lie running, workouts, yoga, etc.)
☐ Diet, food, calorie counter
☐ Weight
☐ Blood pressure
☐ Blood sugar or diabetes
☐ Medication management (tracking, alerts, etc.)
☐ Mood
☐ Sleep
☐ Other (describe below)

11a. Please describe OTHER "apps" you are using for health

____

D. DEVICES

12. Do you use any of the following devices?
(Computer, Tablet computer, Game console, E-book reader, Smartwatch (e.g. Apple, Motorola, Samsung, etc.), Physical activity tracker (e.g: FitBit, Garmin, Misfit, etc.)

◯ Yes
◯ No→ **GO TO QUESTION 13**

12a. If yes please continue .... (check all that apply)

☐ Computer
☐ Tablet computer
☐ Game console
☐ E-book reader
☐ Smartwatch (e.g. Apple, Motorola, Samsung, etc.)
☐ Physical activity tracker (e.g: FitBit, Garmin, Misfit etc.)

E. GAMES

13. Do you play games on your device?

o Yes
o No→ **GO TO QUESTION 14**

13a. What do you play most games on? (check all that apply)

☐ Computer
☐ Tablet
☐ Smartphone
☐ Video game console
☐ Other (describe below)

13b. Please describe OTHER devices ___________________________

14. If we were to develop an electronic Jackson Heart Study mobile application (eJHS app), which would ask health questions periodically, would you be interested in using it?

◯ Yes
◯ No
◯ Not sure/maybe

F. DIGITAL HEALTH TECHNOLOGY

15. Do you use any digital health technology, which store health readings digitally?

○ Yes
○ No → GO TO QUESTION 16

(Digital blood pressure cuff (e.g. Omron, Rite Aid, A&D Medical, etc.)
Digital glucometer (e.g. FreeStyle, Bayer, True Metrix, Accu Check, etc.)
Digital scale (e.g. Taylor, Tanita, Escali, Weight Watchers, etc.)
Other? Please specify

15a. If yes please continue .... (check all that apply)

☐ Digital blood pressure cuff (e.g. Omron, Rite Aid, A&D Medical, etc.)
☐ Digital glucometer (e.g. FreeStyle, Bayer, True Metrix, Accu Check, etc.)
☐ Digital scale (e.g. Taylor, Tanita, Weight Watchers, etc.)
☐ Other? Please specify

15b. Please describe OTHER digital health technology ______________________

F. SOCIAL NETWORKS

16. Do you use a social network to keep in touch with friends and family?

o Yes
o No→ GO TO QUESTION 17
o Prefer not to answer → GO TO QUESTION 17

16a. What social networks do you use? (check all that apply)

☐ Facebook
☐ LinkedIn
☐ Google Plus
☐ Twitter
☐ Other (describe below)

16b. Please describe OTHER social networks that you are using:

____

H. CONTACT INFORMATION

17. May we please have your updated email address where you would prefer to be contacted by the Jackson Heart Study: ___________________________________

18. Will you please list your cell phone? ___________________________________

19. Date Survey Completed ___________________________________
